# Acute quadriparesis revealing Gitelman syndrome: a case report

**DOI:** 10.1097/MS9.0000000000005049

**Published:** 2026-06-10

**Authors:** Harshika Khaim Chandani, Aasiya Ather, Erum Siddiqui, Muhammad Saad Khan, Devya Khaim Chandani, Abubakr Mahmoud

**Affiliations:** a Department of Medicine, Jinnah Sindh Medical University, Karachi, Pakistan; b Department of Medicine, Jinnah Post Graduate Medical Centre (JPMC), Karachi, Pakistan; c Department of Medicine, Shaheed Mohtarma Benazir Bhutto Medical College (SMBBMC); d Department of Medicine, University of Khartoum, Khartoum, Khartoum State, Sudan

**Keywords:** Gitelman syndrome, hypokalemia, hypomagnesemia, metabolic alkalosis, quadriparesis

## Abstract

**Introduction and importance:**

Gitelman syndrome (GS) is a rare autosomal recessive renal tubulopathy caused by SLC12A3 gene mutations, leading to hypokalemia, metabolic alkalosis, hypomagnesemia, and hypocalciuria. Its estimated prevalence ranges from 1 to 10 per 40 000 individuals worldwide, with a carrier frequency approaching 1%, underscoring its potential public health relevance despite underdiagnosis. This case highlights a rare presentation of GS with acute quadriparesis mimicking neurological emergencies, emphasizing the risk of misdiagnosis in acute care settings.

**Case presentation:**

A 30-year-old female presented with acute-onset fever, vomiting, and progressive quadriparesis. Examination revealed flaccid paralysis (power: 2/5), areflexia, and bulbar weakness. Critical biochemical findings included severe hypokalemia (K^+^: 1.6 mmol/l), hypomagnesemia (1.19 mg/dl), metabolic alkalosis (pH: 7.48, HCO_3_^−^: 32 mEq/l), hypocalciuria (6.43 mg/24 h), low urinary potassium, and elevated serum renin.

**Clinical discussion:**

This case demonstrates an intercurrent illness that precipitated acute decompensation in previously undiagnosed GS, leading to profound weakness mimicking Guillain–Barré syndrome, periodic paralysis and Bartter syndrome. The key to diagnosis lies in recognizing the distinctive biochemical triad of hypokalemia, hypomagnesemia, and hypocalciuria with renal potassium wasting. Although genetic testing for SLC12A3 mutations remains the diagnostic gold standard, it is often unavailable in resource-limited settings, making biochemical recognition crucial.

**Conclusion:**

GS is a great mimicker that can present with acute severe paralysis. Clinicians must include it in the differential for unexplained hypokalemia and metabolic alkalosis, as prompt recognition and management with magnesium and potassium supplementation are crucial to prevent life-threatening arrhythmias, recurrent paralysis and reduce long-term morbidity.

## Introduction

Gitelman syndrome (GS) is a rare autosomal recessive salt-losing tubulopathy which is characterized by renal potassium loss, hypokalemia, metabolic alkalosis, hypocalciuria, hypomagnesemia, and hyperreninemic hyperaldosteronism^[^1^]^. This disorder is caused by mutations in the *SLC12A3* gene, located on chromosome 16q13, which encodes the thiazide-sensitive Na–Cl cotransporter (NCC), leading to decreased reabsorption of sodium and chloride in the distal convoluted tubule (DCT) of the kidneys^[^2^]^. To date, more than 500 mutations have been identified, including nonsense, splice-site, and missense mutations that have been linked to GS^[^3^]^. With regard to epidemiology, the prevalence of GS ranges from 1 to 10 instances per 40 000 people and 25 per million, depending on the population being studied. Heterozygous carrier incidence has been reported as up to 1% of the general population, suggesting that GS may be underdiagnosed^[^4^]^. In contrast to the Bartter syndrome, which often appears in infancy, GS is most frequently diagnosed during adolescence or adulthood^[^5^]^. The disease appears to affect both sexes about equally, although some studies indicate a slight predominance in females^[^6^]^.

The clinical spectrum of GS patients is highly variable. Many patients are asymptomatic while others develop symptoms such as muscle weakness, fatigue, tetany, dizziness, vertigo, polyuria, nocturia, and salt craving. In severe cases, recurring muscular paralysis or cardiac arrhythmias have been documented^[^7^]^. These nonspecific symptoms frequently contributes to diagnostic delay or misattribution to more common illnesses, such as hypokalemic periodic paralysis or gastrointestinal potassium losses^[^8^]^. In GS, a comprehensive study of 185 genetically confirmed cases reported specific complication rates: short stature (16.3%), febrile convulsions (13.7%), thyroid dysfunction (4.3%), epilepsy (2.5%), and QT prolongation in one case^[^6^]^. The diagnosis relies on distinctive biochemical abnormalities such as persistent hypokalemia, metabolic alkalosis, hypomagnesemia, and hypocalciuria. It is critical to rule out secondary causes such as diuretic use, vomiting, or unauthorized laxative use. Recent advances in genomes and proteomics have improved understanding of inherited renal tubulopathies like GS. The identification of SLC12A3 mutations by next-generation sequencing allows for early diagnosis, genotype-phenotype association, and family screening. These molecular techniques also aid in personalized therapy approaches. However, due to limited availability in resource-constrained areas, clinical and biochemical criteria are frequently used to make diagnoses^[^9^]^.

HIGHLIGHTS

- *Gitelman syndrome (GS)* is a rare autosomal recessive tubulopathy caused by SLC12A3 mutations, characterized by hypokalemia, hypomagnesemia, hypocalciuria, metabolic alkalosis, and hyperreninemic hyperaldosteronism.
- GS often presents in adolescence or adulthood with variable severity, ranging from asymptomatic electrolyte abnormalities to neuromuscular and cardiac complications.
- The reported case describes a *30-year-old female* presenting with fever and acute quadriparesis, with investigations revealing profound hypokalemia, hypomagnesemia, metabolic alkalosis, and renal potassium wasting consistent with GS.
- Diagnostic challenges include overlap with hypokalemic periodic paralysis, gastrointestinal potassium losses, and neuromuscular disorders, especially in *resource-limited settings where genetic testing is unavailable*.
- Management involves *potassium and magnesium supplementation*, potassium-sparing diuretics, and dietary modifications, with early recognition crucial for preventing recurrent paralysis, arrhythmias, and long-term complications.

The primary treatment for GS is to correct electrolyte imbalances, provided there are no significant complications of the disease. This involves potassium and magnesium supplementation to address the disease’s deficits. Potassium-sparing diuretics, such as spironolactone or amiloride, can help minimize potassium loss^[^10^]^. In addition, a high-salt diet is often advised to compensate for urinary sodium loss. Early diagnosis and management of GS are crucial for preventing complications, enhancing patients’ quality of life, and reducing recurrent hospitalizations due to severe electrolyte imbalances. Due to its rare and varying appearance, GS remains a diagnostic challenge. Case reports are crucial for emphasizing atypical presentations, extending the clinical spectrum, and leading physicians to timely detection. We discuss the case of a young woman with acute quadriparesis and biochemical characteristics suggestive of GS. This example highlights the need for investigating GS in patients who present with unexplained electrolyte imbalances and adds to the little regional literature on this condition. This case report has been written in compliance with the CARE guidelines and checklist for case reports^[^11^]^.

## Case presentation

A 30-year-old married female, with no known comorbidities, presented to the Emergency Department with a 3-day history of fever and 2 days of progressive weakness affecting all four limbs. She was well until 3 days prior to admission when she developed a sudden onset of high-grade continuous fever, occurring throughout the day and relieved with the antipyretics. This was accompanied by nausea and projectile vomiting for 1 day; the vomitus was watery, contained food particles, and was non-bilious and non-bloodstained. There was no history of chills, rigor, rash, seizures, headache, sore throat, retro-orbital pain, chest pain, cough, palpitations, abdominal pain, jaundice, diarrhea, night sweats, weight loss, tuberculosis contact, joint pain, or urethral discharge. She also had no recent travel or pet exposure. Two days before the presentation, she developed sudden-onset weakness of all four limbs, initially starting in the left lower limb and then progressing to the right lower limb and upper limbs. The weakness was more marked in the lower extremities and led to inability to walk or to perform activities of daily living. It was associated with progressive shortness of breath corresponding to MMRC (Modified Medical Research Council) class III–IV. There was no history of chest pain, orthopnea, paroxysmal nocturnal dyspnea, abdominal distension, pedal edema, frothy urine, trauma, vertigo, seizures, paresthesia, diplopia, dysphagia, visual changes, headache, oral ulcers, photosensitivity, alopecia, rash, joint pain, cyanosis of digits, palpitations, thyroid-related symptoms or urinary/fecal incontinence. Her history was unremarkable except for a cesarean section 2.5 years ago, during which she received two pints of packed red cells. There was no significant drug history or recent travel. She reported adequate dietary intake of vegetables, pulses, chicken, and red meat. Personal history revealed disturbed sleep and decreased appetite with normal bowel and bladder habits and no addictions reported. Menstrual cycles were regular (5 days/28 days) with dysmenorrhea but no heavy menstrual bleeding, dyspareunia or post-coital bleeding. Obstetric history included one intrauterine death and one healthy pregnancy that was delivered via cesarean section 2.5 years ago. Both parents and four siblings were alive and healthy with no family history of tuberculosis. Socioeconomically, she belonged to a middle-class family living in a well-ventilated two-room house shared by four members, consuming tap water and with no pet exposure.

**Figure 1.**
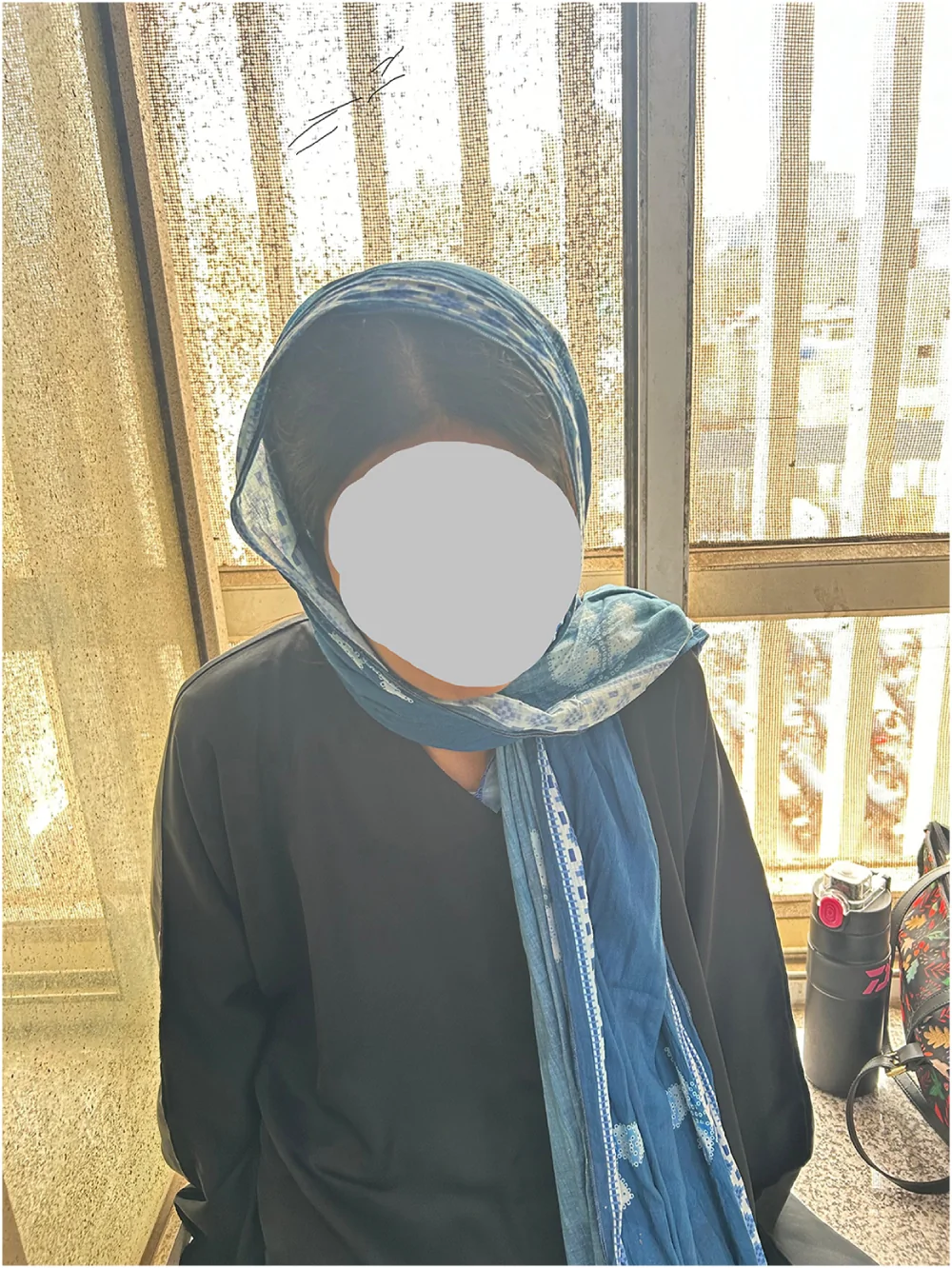
Stepwise clinical diagnostic pathway for Gitelman syndrome in resource-limited settings.

On examination, she was of average build, alert and oriented to time, place, and person. Blood pressure was 90/60 mm Hg, pulse 70 beats per minute, respiratory rate 24 breaths per minute, temperature 100 °F, and oxygen saturation 97% on room air. She appeared anemic and dehydrated but there was no jaundice, clubbing, cyanosis, edema, or lymphadenopathy (Fig. 1). Jugular venous pressure was not raised. Neurological assessment revealed a Glasgow Coma Scale score of 15/15. Pupils were equal and reactive, and muscle bulk and tone were preserved but power was reduced to 2/5 in all limbs with absent reflexes. Plantar responses were bilaterally mute, while sensations, joint position sense, and proprioception remained intact. No fasciculations were observed. Extraocular movements, fundoscopy, and facial sensation and symmetry were normal but palatal movements were reduced and the patient was unable to cough effectively. Cerebellar signs and gait could not be assessed as she was unable to sit, stand, or walk. Systemic examination was otherwise unremarkable. The abdomen was scaphoid, non-tender with no visceromegaly or ascites. Cardiovascular examination showed a normally placed apex beat with audible S1 and S2 and no added sounds. Respiratory assessment revealed symmetrical chest movements, centrally placed trachea, resonant percussion note, and normal vesicular breath sounds bilaterally without additional sounds. Investigations included a chest radiograph, which was normal with clear lung fields and visible costophrenic angles. CT and MRI of the brain, EEG, and cerebrospinal fluid studies were unremarkable. MRI of the cervical and dorsal spine revealed no abnormality. Baseline workup including complete blood count, liver function tests, urine detailed report, antinuclear antibody profile, and transthoracic echocardiography did not reveal any significant abnormalities (Table 1). Blood culture was positive for *Staphylococcus* species (non-aureus); however, in the absence of clinical features of sepsis and with subsequent clinical stability, this was considered likely a contaminant. Viral serology for HBsAg, anti-HCV, and HIV was non-reactive. Laboratory analysis revealed severe hypokalemia (1.6 mEq/l; reference 3.5–5.0 mEq/l), hypomagnesemia (1.19 mg/dl; reference: 1.7–2.2 mg/dl), and hypocalcemia (6.73 mg/dl; reference: 8.5–10.5 mg/dl). Arterial blood gas showed metabolic alkalosis (pH 7.48; reference 7.35–7.45, HCO_3_^−^ 32 mEq/l; reference: 22–28 mEq/l, PCO_2_: 45 mm Hg). Serum osmolality was 285 mOsm/kg (reference: 275–295 mOsm/kg) and urine osmolality was 276 mOsm/kg. Urinary studies demonstrated elevated urinary sodium (110 mmol/l), low urinary chloride (108 mmol/l), and urinary potassium (14.8 mmol/l). Twenty-four-hour urinary calcium was low (6.43 mg/24 h; reference 100–300 mg/24 h), confirming hypocalciuria. Serum renin was elevated (55.9 uIU/ml), supporting hyperreninemic salt wasting (Tables 2 and 3). Serum aldosterone levels were not available at our center due to logistical constraints. However, the presence of elevated renin levels in the setting of normotension, metabolic alkalosis, hypokalemia, hypomagnesemia, and hypocalciuria was considered sufficient to support a diagnosis of GS. On ABGs blood pH was 7.48, HCO_3_^−^ was 32 mEq/l and PCO_2_ was 45 mm Hg. The anemia was normocytic and normochromic and was considered likely secondary to nutritional factors, as no evidence of hemolysis or active blood loss was identified.

**Table 1:** Complete blood count.

Parameter	Value	Normal range
Hb (g/dl)	8.7	Male: 13.8–17.2/Female: 12.1–15.1
MCV (fl)	85.3	80–100
RBC (mill/µl)	3.02	Male: 4.7–6.1/Female: 4.2–5.4
TLC (×10^9^/l)	11.1	4.0–11.0
Neutrophils (%)	86.2	40–70
Lymphocytes (%)	9.7	20–40
Platelet count (×10^9^/l)	302	150–450

**Table 2:** Urea, creatinine, and serum electrolytes.

Parameter	Test value	Normal range	Unit
Urea	21	7–20 mg/dl	mg/dl
Creatinine	0.5	0.6–1.3 mg/dl	mg/dl
Sodium (Na)	140	136–145 mmol/l	mmol/l
Potassium (K)	1.6*	3.5–5.1 mmol/l	mmol/l
Chloride (Cl)	113	98–107 mmol/l	mmol/l
Calcium	6.73	8.6–10.3 mg/dl	mg/dl
Magnesium	1.19	1.6–2.6 mg/dl	mg/dl
Phosphorous	1.49	2.5–4.5 mg/dl	mg/dl

To further evaluate hypokalemia, the transtubular potassium gradient (TTKG) was calculated using the patient’s serum and urine values (serum potassium: 1.4 mEq/l, urine potassium: 14.8 mmol/l, serum osmolality: 285 mOsm/kg, urine osmolality: 276 mOsm/kg). The calculated TTKG was 10, which is inappropriately elevated in the setting of hypokalemia and indicates ongoing renal potassium wasting. This finding, in combination with hypomagnesemia, hypocalciuria, metabolic alkalosis, and elevated renin levels, was strongly suggestive of GS. Continuous cardiac monitoring was performed during electrolyte correction. No clinically significant arrhythmias were observed.

## Management

**Figure 2.**
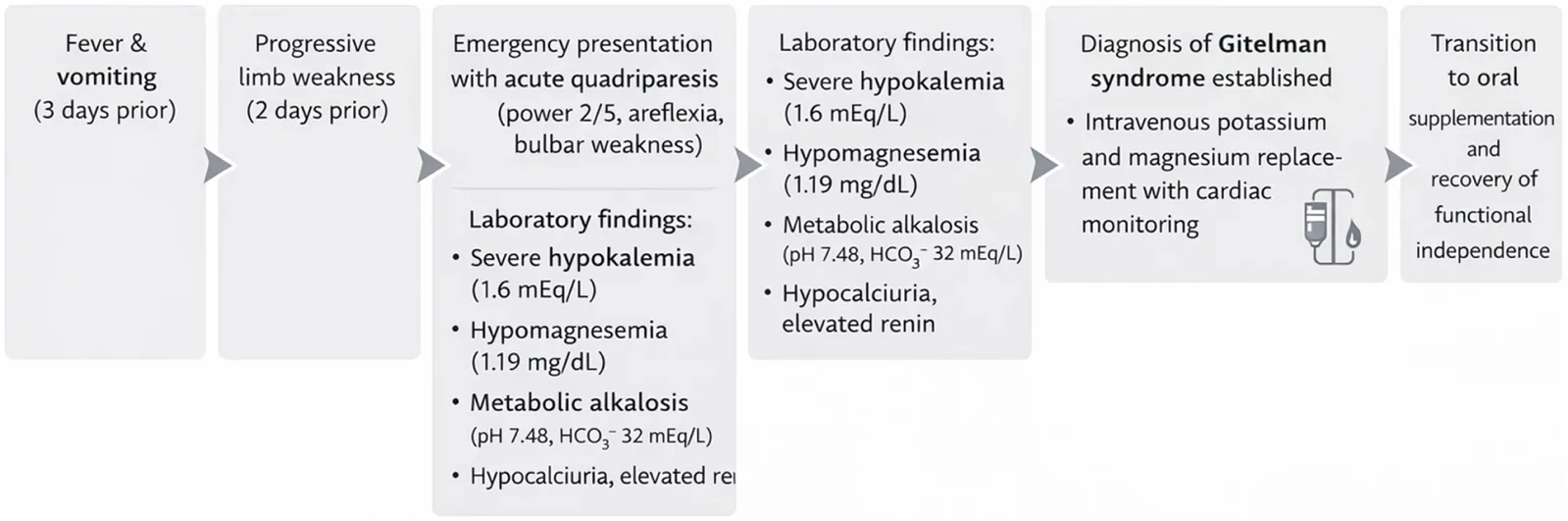
Physical appearance.

The patient was managed with intravenous potassium chloride infusion (20 mEq diluted in 100 ml normal saline over 1 hour, repeated as required with cardiac monitoring) along with intravenous magnesium sulfate replacement followed by transition to oral potassium and magnesium supplementation. A liberal salt diet was advised. Potassium-sparing agents were considered; however, electrolyte levels improved satisfactorily with supplementation alone during hospitalization. Figure 2 illustrates the patient’s clinical timeline, summarizing the onset of symptoms, progression of weakness, laboratory findings, diagnosis of GS, management with electrolyte replacement, and gradual recovery of functional independence.

**Figure 3.**
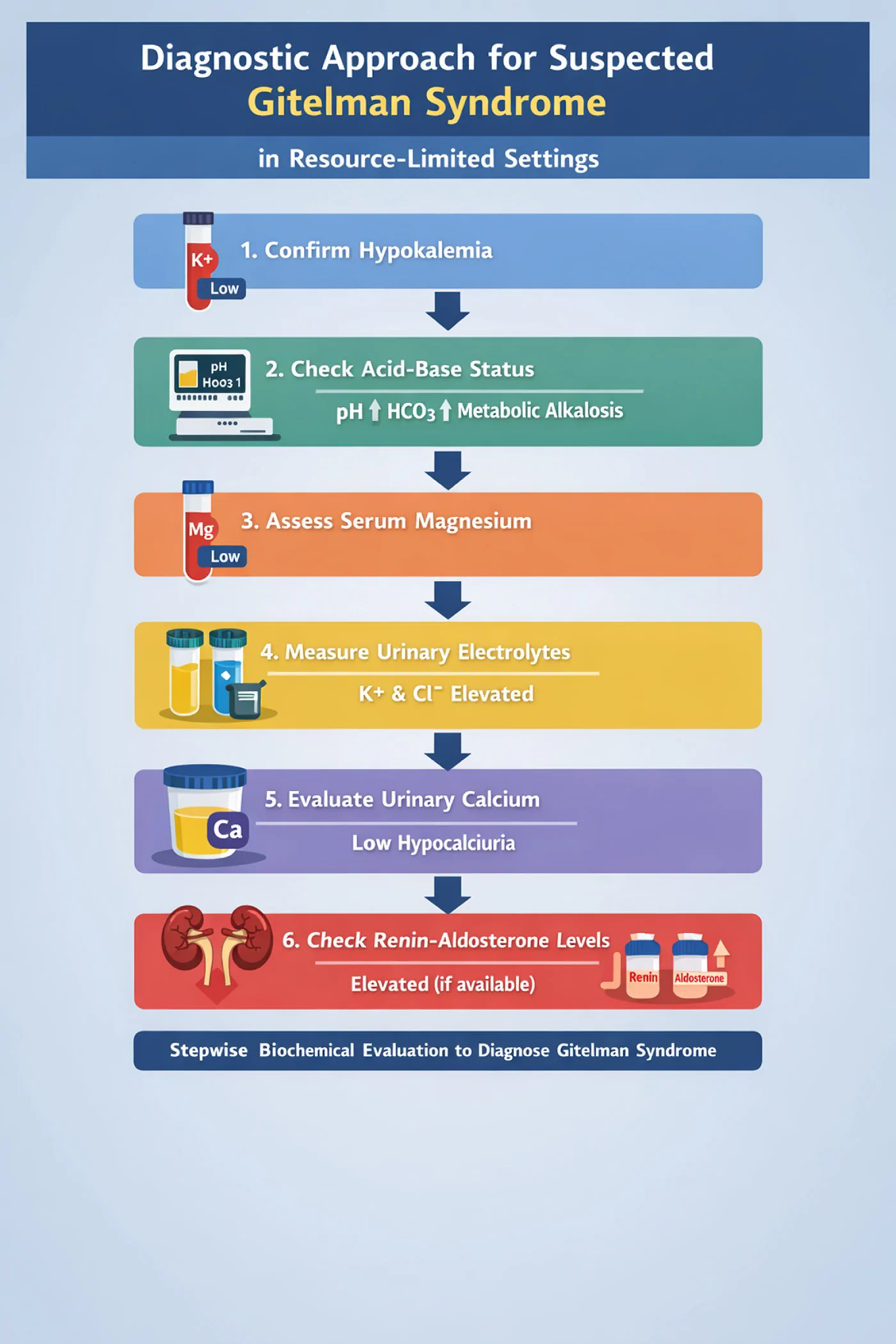
Clinical timeline of the GS patient.

The main differentials considered included hypokalemic periodic paralysis, Guillain–Barré syndrome with bulbar involvement, transverse myelitis, HIV-associated neuropathy, Lyme disease, myasthenia gravis, tick paralysis, and thyrotoxic periodic paralysis. However, the diagnosis of GS was established based on the presence of metabolic alkalosis, hypocalciuria, hypokalemia with renal potassium and chloride wasting and hypomagnesemia, elevated renin levels, and compatible clinical features of generalized weakness, muscle fatigue, cramps, and limb weakness.

## Discussion

GS is an autosomal recessive tubulopathy that was first described in 1966. It is characterized by hypokalemic metabolic alkalosis, hypocalciuria, hypomagnesemia, and elevated renin and aldosterone levels in the absence of hypertension^[^12^]^. It results from inactivating mutations in the *SLC12A3* gene encoding the thiazide-sensitive sodium-chloride cotransporter (NCC) in the distal convoluted tubule (DCT). Impaired sodium reabsorption in the DCT leads to compensatory activation of the renin-angiotensin-aldosterone system (RAAS) with subsequent renal potassium and magnesium wasting^[^13^]^. It is often considered a benign salt-wasting disorder but GS may present with significant clinical heterogeneity ranging from asymptomatic electrolyte abnormalities to profound neuromuscular and cardiac complications^[^14^]^. The present case highlights a diagnostically challenging presentation of GS in a 30-year-old female patient who developed acute quadriparesis following a febrile illness which underscores the importance of recognizing this syndrome in the differential diagnosis of unexplained hypokalemic paralysis. Although GS is considered less severe than Bartter syndrome, its clinical spectrum is remarkably broad^[^15^]^. Many patients suffering from GS remain asymptomatic and are diagnosed incidentally on biochemical testing, whereas others develop significant muscle weakness, cramps, tetany, salt craving, dizziness, polyuria and nocturia^[^6^]^. Its severe manifestations include recurrent paralysis and cardiac arrhythmias that have been documented but are rare^[^16^]^. In a review of 185 genetically confirmed cases, complication rates included short stature (16.3%), febrile convulsions (13.7%), thyroid dysfunction (4.3%), epilepsy (2.5%) and QT prolongation in one case^[^6^]^. These findings emphasize that GS can no longer be regarded as a purely benign disease but rather one with potentially disabling or life-threatening consequences. The nonspecificity of presenting symptoms makes the diagnosis more challenging. In usual clinical practice, hypokalemic paralysis is more commonly attributed to causes such as gastrointestinal potassium loss, diuretic abuse, thyrotoxic periodic paralysis, or neuromuscular disorders^[^17^]^. In our patient, the acute onset of flaccid quadriparesis initially raised concerns for Guillain–Barré syndrome, transverse myelitis and myasthenia gravis but normal cerebrospinal fluid, neuroimaging and electrophysiological studies ruled out these differentials. Recognition of the biochemical triad of hypokalemia, metabolic alkalosis, and hypomagnesemia proved decisive in guiding the diagnosis toward GS. Another diagnostic pitfall arises from the overlap with Bartter syndrome, which is also a similar salt-losing tubulopathy. Distinguishing features include the typical onset in adolescence or adulthood, absence of hypercalciuria and relative preservation of growth in GS whereas Bartter syndrome usually manifests in infancy with polyhydramnios, growth retardation and significant hypercalciuria^[^18^]^. Our patient’s presentation in adulthood, normal stature, and low urinary calcium favored our diagnosis of GS.

The biochemical abnormalities explain the neuromuscular manifestations seen in GS. Hypokalemia reduces resting membrane potential, rendering muscles less excitable and predisposing to weakness, cramps and paralysis. Concurrent hypomagnesemia further exacerbates neuromuscular irritability, tetany and in severe cases, seizures^[^19^]^. Cardiac arrhythmias that include QT prolongation and torsades de pointes have also been reported. This is reflecting the combined effects of hypokalemia and hypomagnesemia on myocardial excitability. Our patient developed acute quadriparesis precipitated by fever and vomiting. Intercurrent illness, increased urinary losses, and decreased intake likely aggravated her underlying electrolyte derangements, tipping a compensated state into symptomatic decompensation. This phenomenon underscores the dynamic nature of GS in which patients may remain clinically stable until additional stressors provoke acute manifestations. The estimated prevalence of GS is between 1 and 10 per 40 000, with a carrier frequency of up to 1% in the general population^[^7^]^. However, true prevalence may be underestimated due to the high proportion of asymptomatic or misdiagnosed cases. In resource-limited settings where genetic testing is not widely available, the diagnosis relies heavily on biochemical profiles, which can be confounded by secondary causes of electrolyte imbalance. In South Asia, data on GS are scarce, with most literature limited to isolated case reports. Our case thus adds to the regional documentation of GS, emphasizing the need for heightened clinical suspicion. Considering the high burden of diarrheal disease, malnutrition, and indiscriminate use of diuretics and laxatives in the local population, GS may often be overlooked.

The differential diagnosis of hypokalemic paralysis includes a wide spectrum of conditions. Hypokalemic periodic paralysis which is either familial or thyrotoxic and is characterized by episodic weakness but lacks persistent metabolic alkalosis, hypomagnesemia, and renal potassium wasting^[^20^]^. Gastrointestinal potassium loss from vomiting or diarrhea can mimic GS biochemically but urine potassium and chloride are low in these cases but they are elevated in GS. In our patient, hypokalemic periodic paralysis was initially suspected given the acute onset of weakness but laboratory investigations showing metabolic alkalosis, hypomagnesemia, elevated urinary sodium, potassium and chloride excretion along with suppressed calcium excretion clarified the diagnosis of GS. Guillain–Barré syndrome and myasthenia gravis were also considered but excluded through normal cerebrospinal fluid analysis, imaging, and electrophysiological studies.

Genetic confirmation of *SLC12A3* mutations remains the gold standard for diagnosis. Over 500 mutations including missense, nonsense and splice-site variants have been described^[^21^]^. Genetic testing provides definitive diagnosis and aids in family counseling but its availability and affordability remain limited in many parts of the world including Pakistan. Consequently, clinicians must rely on careful interpretation of clinical and biochemical findings. Our case demonstrates that even in the absence of molecular confirmation, a confident diagnosis of GS can be achieved through characteristic biochemical patterns. A stepwise clinical diagnostic pathway for GS in resource-limited settings is illustrated in Figure 3.

Management of GS is largely supportive and aimed at correcting electrolyte disturbances. Potassium and magnesium supplementation form the cornerstone of therapy^[^22^]^. Magnesium replacement is particularly challenging due to poor gastrointestinal absorption and frequent gastrointestinal side effects which necessitate high doses or slow-release formulations. Potassium-sparing diuretics such as spironolactone, eplerenone, or amiloride can help mitigate renal potassium loss by antagonizing aldosterone effects in the distal nephron^[^23^]^. A liberal salt diet is generally advised to offset chronic urinary sodium loss. Early recognition of GS has practical clinical implications. Prompt diagnosis reduces unnecessary neuroimaging, invasive procedures, and prolonged hospitalization. Timely electrolyte correction may shorten hospital stay, decrease healthcare costs, and prevent life-threatening complications such as cardiac arrhythmias and renal compromise. Our patient was managed with intravenous potassium and magnesium supplementation which was followed by oral replacement and dietary advice. Her weakness gradually improved and she regained functional independence. Long-term follow-up is essential to ensure electrolyte stability, adherence to therapy and monitoring for complications such as arrhythmias or nephrocalcinosis.

The overall prognosis of GS is favorable compared with other tubulopathies, provided the electrolyte imbalances are adequately managed. Nonetheless, recurrent symptoms, hospitalization for acute paralysis and long-term complications such as chondrocalcinosis, cardiac arrhythmias, and nephrolithiasis can significantly affect quality of life. Furthermore, hypomagnesemia may also predispose to insulin resistance, osteoporosis and vascular dysfunction which necessitate the ongoing vigilance. For women of childbearing age, GS poses additional challenges during pregnancy, when physiological changes in renal handling of electrolytes may exacerbate hypokalemia and hypomagnesemia^[^24^]^. Close monitoring and tailored supplementation are crucial to minimize maternal and fetal risks.

This case illustrates several key aspects of GS. At first, it highlights the importance of considering GS in the differential diagnosis of hypokalemic paralysis particularly in adult patients with metabolic alkalosis and hypomagnesemia. Secondly, it underscores the potential for intercurrent illness to precipitate acute decompensation in otherwise stable patients. Thirdly, it demonstrates the feasibility of making a clinical diagnosis in resource-limited settings where genetic testing is unavailable. Finally, it emphasizes the need for long-term management strategies aimed not only at electrolyte replacement but also at prevention of complications and improvement of quality of life.

Regarding its uniqueness, Table 4 summarizes recent case reports (2023–2025) of GS presenting with acute neuromuscular features, predominantly in male patients who presented with weakness mimicking conditions like Guillain-Barré syndrome and improved with electrolyte correction. Our case of a 30-year-old female with acute quadriparesis aligns with these reports but presents distinctive features, including its occurrence in a female patient and the severe, acute decompensation (power 2/5 with bulbar weakness) clearly precipitated by a febrile illness. Furthermore, despite the unavailability of genetic testing, our case establishes a robust clinical diagnosis using the classic biochemical triad and pathognomonic hypocalciuria, reinforcing that a confident diagnosis of GS is achievable even in resource-limited settings. The interpretation of the TTKG in this case may be limited, as the calculation can be unreliable when urine and plasma osmolality are nearly equal. Therefore, TTKG results should be interpreted with caution.

**Table 3:** Urinary electrolyte analysis.

Parameter	Patient value	Normal range	Unit
Urinary sodium	110	40–220	mmol/l
Urinary potassium	14.8	25–125	mmol/l
Urinary chloride	108	110–250	mmol/l
Urinary calcium	6.43	100–250	mg/24 h*

**Table 4:** Summary of the reported cases of Gitelman Syndrome with acute neuromuscular features (2023–2025).

Author (year) [reference]	Age/Sex	Trigger/Presentation	Key biochemical findings	Mimicked condition/feature	Outcome
Rocha *et al* (2023)^[^25^]^	21-year-old male	General muscle weakness & myalgia	Hypokalemia, hypomagnesemia, metabolic alkalosis	Generalized muscle weakness without specific paralysis	Improved with management
Gunde *et al* (2023)^[^26^]^	Middle-aged male	Acute limb weakness progressing over 8 days	Hypokalemia, hypomagnesemia, metabolic alkalosis, hypocalcemia	Acute hypokalemic paralysis initially misdiagnosed as Guillain-Barré syndrome	Improved with electrolyte correction
Loni *et al* (2024)^[^27^]^	10-year-old male	Acute infection + muscle weakness (failure to thrive)	Hypokalemia, hypomagnesemia, metabolic alkalosis	Generalized muscle weakness in pediatric GS	ICU support & recovery
Gulve *et al* (2024)^[^28^]^	Adult (exact age not specified)	Acute hypokalemic periodic paralysis	Hypokalemia	Hypokalemic periodic paralysis	Recovery with supplementation
Teir *et al* (2025)^[^29^]^	17-year-old male	Progressive lower limb paralysis	Severe hypokalemia, hypomagnesemia, metabolic alkalosis, hypocalciuria, hyperreninemia	Recurrent hypokalemic periodic paralysis	Full recovery after aggressive correction

The differential diagnosis of hypokalemic metabolic alkalosis encompasses several renal and extrarenal causes. A comparative summary of key distinguishing features between GS, Bartter syndrome, vomiting-induced chloride depletion, diuretic abuse, and thyrotoxic periodic paralysis is provided in Supplemental Digital Content Table 1, available at: http://links.lww.com/MS9/B190. The presence of hypomagnesemia, hypocalciuria, elevated urinary chloride, and hyperreninemia in our patient strongly favored GS over other etiologies.

## Conclusion

GS, though rare, should be considered in patients presenting with unexplained hypokalemia, metabolic alkalosis, and hypomagnesemia. Its heterogeneous clinical manifestations and biochemical overlap frequently mimic neurological and metabolic disorders, leading to diagnostic delay. Our case of a 30-year-old woman with acute quadriparesis highlights the potential severity of GS and reinforces the role of careful biochemical evaluation in establishing the diagnosis. Early identification and aggressive correction of electrolyte abnormalities can prevent arrhythmias, recurrent paralysis, and long-term renal sequelae. Greater awareness among clinicians, coupled with regional reporting of cases, is essential to address the underdiagnosis of this condition and to optimize care in resource-constrained settings.

## Data Availability

All data generated or analyzed during this study are included in this published article.
